# Re-envisioning, Retooling, and Rebuilding Prevention Science Methods to Address Structural and Systemic Racism and Promote Health Equity

**DOI:** 10.1007/s11121-022-01439-4

**Published:** 2022-10-12

**Authors:** Velma McBride Murry, Cory Bradley, Gracelyn Cruden, C. Hendricks Brown, George W. Howe, Martín-Josè Sepùlveda, William Beardslee, Nanette Hannah, Donald Warne

**Affiliations:** 1 Departments of Health Policy & Human and Organizational Development, Vanderbilt University, Nashville, TN USA; 2 Washington University School of Medicine in St. Louis, St. Louis, MO USA; 3 Oregon Social Learning Center, Eugene, OR USA; 4 Northwestern University Feinberg School of Medicine, Chicago, IL USA; 5 George Washington University, Washington, DC USA; 6 Florida International University, Miami, FL USA; 7 Harvard Medical School, Boston Children’s Hospital, Judge Baker Children’s Center, Boston, MA USA; 8 Center for Indigenous Health, Johns Hopkins University, Baltimore, MD USA

**Keywords:** Systemic and structural racism, Social justice, Prevention Science, Community voices and engagement, Representational equity

## Abstract

The historic momentum from national conversations on the roots and current impacts of racism in the USA presents an incredible window of opportunity for prevention scientists to revisit how common theories, measurement tools, methodologies, and interventions can be radically re-envisioned, retooled, and rebuilt to dismantle racism and promote equitable health for minoritized communities. Recognizing this opportunity, the NIH-funded Prevention Science and Methodology Group (PSMG) launched a series of presentations focused on the role of Prevention Science to address racism and discrimination guided by a commitment to social justice and health equity. The current manuscript aims to advance the field of Prevention Science by summarizing key issues raised during the series’ presentations and proposing concrete research priorities and steps that hold promise for promoting health equity by addressing systemic racism. Being anti-racist is an active practice for all of us, whether we identify as methodologists, interventionists, practitioners, funders, community members, or an intersection of these identities. We implore prevention scientists and methodologists to take on these conversations with us to promote science and practice that offers every life the right to live in a just and equitable world.

## Introduction

Whether expressed as subtle or overt discrimination and oppression, systemic racism has been and continues to be a primary source of stress for Black, Indigenous, Pacific Islander, Latino/a/x Hispanic, and Asian (BIPILA) peoples (Beard et al., 2022). By systemic racism, we refer to the societal condition wherein advantages, opportunities, and value are structurally allocated based on race and ethnicity through collective practices, mechanisms, behaviors, and beliefs enacted through social systems, in such a way, that reproduces and maintains racial hierarchies. These structural and systemic processes ultimately are manifested as racial hierarchy and domination of whiteness, even without much thought or intent. The term “systemic” alludes to the reproduction and reification of that racial hierarchy and white dominance in “whole systems” including their structures (laws, policies, norms, institutional practices), which comprise and uphold them (Bonilla-Silva, 2021; Braveman et al., 2022; Jones, 2008).

For Black Americans, systemic racism has been characterized as a “ubiquitous, continuous, contextual variable that continues to impact every aspect of their everyday life” (Murry et al., 2001, p. 917; also see Murry et al., 2018; Williams & Mohammed, 2013). Racial/ethnic disparities in COVID-19 infections and deaths sparked national conversations about the implications of systemic racism as a key driver of social determinants of health that disproportionately affect minoritized populations. These conversations not only acknowledged the pervasive nature of systemic racism but heightened awareness of the urgency to dismantle patterns of discrimination and oppression. Like other social ills, those from minoritized groups were at higher risk of COVID-19 exposure because they disproportionately represented essential workers and lacked equitable access to resources that could mitigate exposure risk (Barkin et al., 2020). The perpetually damaging nature of systemic racism is also conveyed through social media coverage of economic and political oppression experienced by minoritized communities at the hands of the “laws, written or implicit policies, and entrenched practices and belies that produce, condone and perpetuate widespread unfair treatment” [and marginalization] (Braveman et al., 2022, p. 171). Systemic racism creates a pervasive sense of fear and terror that impact every aspect of minoritized families’ lives, with implications for their health and safety. Reasons for public safety concerns are warranted given increasing reports of fatal encounters of Black people with police officers and White vigilante violence (Fridkin et al., 2017).

The environments and conditions created and sustained by systemic racism have been characterized metaphorically as toxic, hazardous, polluted waters Black families swim in as they navigate everyday life experiences (Murry, 2019). These toxic environments have immediate and long-term consequences for morbidity and mortality in minoritized populations—impeding health-promoting behaviors, academic performance, emotional health, and physical health (Morsy & Rothstein, 2019). They are associated with early onset of chronic diseases (Brody et al., 2016) and subsequent early death (Williams & Mohammed, 2013).

It is noteworthy that toxic environments are creations of broader social, economic, educational, political, and legal conditions. Characterized as social determinants of health, they manifest as disparities in a wide range of health outcomes. This involves disparities in behavioral risk factors, environmental exposures, and the quality of and access to health care and preventive intervention programming. They occur in multiple domains including health, housing, income, employment, and education (Braveman & Gottlieb, 2014).

Thus, the recent alarms raised in the USA regarding the need to address systemic racism hearken to the urgency expressed by Dr. Martin Luther King, Jr., who in 1965 stated that “Of all the forms of inequality, injustice in health is the most shocking and the most inhumane.” These words frame a contemporary call to action for the field of Prevention Science. They press prevention scientists to engage with the present-day momentum of national conversations on the roots and current impacts of racism in the USA and revisit how common theories, measurement tools, methodologies, and interventions can be radically re-envisioned, retooled, and rebuilt to dismantle systemic and structural racism and promote equitable health for minoritized communities.

Centering Prevention Science on equitable outcomes and anti-racism requires consciously and directly confronting the disparate health outcomes created by the consequences of systemic racism, manifested as toxic upstream sources that have created and sustained institutionalized inequities in research development (i.e., knowledge production), training, policies, and practice. Upstream toxic waters flow downstream and filter into every fabric of our society. These waters, intentionally and unintentionally, facilitate rivers of advantage and privilege for racially identified White communities to systematically experience greater social, political, and economic access. Other rivers and lakes emit toxins that flow from systemic racism and discrimination to produce and sustain situations and conditions that hinder opportunities for Black, Indigenous, Pacific Islander, Latino/a/x, Hispanic, and Asian (BIPILA) communities to be as healthy as possible (Thornton et al., 2016). As noted in the prior Commission on the Social Determinants of Health (CSDH, 2008) report, “This unequal distribution is not in any sense a “natural” phenomenon but is the result of a toxic combination of poor social policies and programs, unfair economic arrangements, and bad politics. Together, the structural determinants and conditions of daily life constitute the social determinants of health and are responsible for a major part of health inequities between and within countries” (Cp. 1).

In lieu of revisiting the dire statistics and cogent analyses that several esteemed scholars have labored to produce concerning racialized health disparities (Williams & Mohammed, 2013), we seek to discursively engage the field of Prevention Science concerning the myriad opportunities to disrupt systemic racism and advance health equity by evaluating our role as prevention scientists to consider: (1) “Are we implicitly or explicitly perpetuating social injustices and inequity?”; and, (2) “What can we do to promote and facilitate social justice and health equity?” Addressing these questions requires reflective evaluation of the field of Prevention Science. We need to critically examine when and how current theories, measurement tools, methodologies, and the design, development and testing of preventive interventions may perpetuate injustice, and how they can be radically re-envisioned, retooled, and rebuilt to dismantle racism and promote equitable health for minoritized communities.

Our hope is that this contribution will stimulate the field of Prevention Science to be *willing* to not only critically evaluate principles that guide and inform our work but to be *bold* enough to accept that foundational principles of Prevention Science may in fact be based on historical legacies that perpetuate systemic racism and social injustice. Such knowledge, we hope, will lead to the creation of new paradigms, refined conceptual frameworks and theories, and new measurement tools and implementation strategies, guided by the goals of health equity and social justice. These radical changes have implications for transforming scientific policies and practices, and, most importantly, leading to improved health for all persons regardless of their race or social class.

## Conversations Towards a Paradigmatic Shift in Prevention Science

This commentary was convened through leveraging insights shared during a recent Prevention Science and Methodology Group (PSMG) series of presentations and discourses focusing on the capacity of Prevention Science to address health disparities that are consequent to racism and discrimination and on efforts to center social justice and health equity in Prevention Science. We summarize key issues raised during the series’ presentations and propose concrete examples of how to advance a paradigm shift in the field of Prevention Science to promote social justice and health equity by deliberately addressing systemic racism. While the original focus of our series was on the need to address racial justice for Black Americans who have been historically marginalized, colonized, and discriminated against in the USA (Hoppe et al., 2019), we also engaged scholars whose work examined ways in which oppression and injustice affect the lives of Indigenous, Pacific Islander, Latino/s/a/a, and Asian families, children, and communities (Beard et al., 2022), including presentations that applied intersectionality as a theoretical framework to inform and guide preventive interventions.

## Revising Use of Theoretical Frameworks in Prevention Science

Rising to meet the public health and ethical threat of systemic racism invites an opportunity to reimagine Prevention Science as a field anchored in equity. It calls for critically questioning the principles upon which we conduct and report Prevention Science, through deconstructing and reconstructing both theory and research in ways that actively address racialized health inequities and unjustly distributed health determinants at multiple levels of influence. One such approach for advancing Prevention Science by embedding culturally relevant and critical systems theories to existing frameworks was offered by two PSMG presenters, Doucet and Supplee, who prompted attendees to consider what policy, practice, and prevention science would look like if truly guided by critical race theory, for example (Supplee & Doucet, 2021). As Doucet wrote in a related blog post, “Critical theories interrogate societal systems and structures with particular attention to the production and reproduction of power hierarchies, social inequities, and the taken-for-granted assumptions of ideological hegemonies, such as settler colonialism, patriarchy, white supremacy, capitalism, and imperialism.”

Doucet proposed that anti-racism research is responsive (centers diverse voices and equity during research question generation which we discuss later), routinized or iterative contextual inquiry (asks racism-conscious questions in multiple phases of research investigation), and relational (considers how power dynamics affect trust among stakeholders, and who gets to engage with decision-makers). As an example, Supplee and Doucet pointed to the work of Ferdinand et al. (2017) who worked within organizational structures to reduce racism against Aboriginal communities in Australia rather than working with Aboriginal communities to cope with racism. These examples, in addition to Murry and colleagues’ theoretical model (2018), offer insights on why (theoretically) and how (methodologically) systemic racism can and should be directly addressed and embraced within Prevention Science. This includes expanding our focus on problem-centered approaches to also include protective processes.

## Risk and Protective Mechanisms

A recent review of NIH-funded prevention research projects points to a paucity of relevant research on the role of social factors as increasing or protecting against risk for health disparities (Hoppe et al., 2019). This deficit is particularly disturbing given that general socioeconomic and race/ethnicity factors have been found to explain 60% of the variation in life expectancy among US counties (Dwyer-Lindgren et al., 2017). This void in evidence suggests the need for a proliferation of diverse, critical theories and methods enabling Prevention Science to pinpoint mechanisms by which systemic racism impacts equitable health outcomes among racial and ethnic populations. For example, Hardeman et al. (2021) creatively integrated evidence to demonstrate that residing in high police contact neighborhoods was a leading predictor of preterm birth among Black individuals in Minneapolis. In addition, results from Sewell’s (2016) exploratory analysis revealed linkages among dual mortgage market political economies, ethno-racial residential segregation at the neighborhood level, and childhood health inequalities at the microlevel. Both studies highlight larger macro-level social system factors that may need to be targeted in preventive intervention trials to demonstrate stronger effects. Mechanisms at the macro-level can be among the most impactful, significant upstream intervention targets to prevent or avert leading risk factors associated with health disparities outcomes.

Prevention research that advances health equity includes studies that investigate leading risk factors for death and disability among marginalized populations. Those risk factors contribute to the body of evidence that demonstrates the impacts of structural racism on health outcomes; moreover, inclusion of a more diverse body of evidence pointing to macrolevel risk factors would be essential in providing clear explanations of the mechanisms by which systemic racism impacts the health of marginalized communities.

## Designing and Testing Upstream Prevention Interventions

Revisiting and rebuilding preventive interventions to address racial equity will require adapting and refining concepts and methodological approaches to increase relevance, acceptability, and appropriateness of interventions. Yet, the interventions with the deepest and broadest population health impact will likely require entirely unique interventions to address not only downstream problems, but upstream and middle stream structural barriers that create and maintain inequities and injustices. We propose that the field of Prevention Science promote the development and evaluation of interventions that target institutional racism in our institutions, such as public and professional schools, health organizations, and the justice system. For example, Hardeman et al. (2022) noted the disconnect between conceptualization and operationalization of systemic racism. It is part of a system, interconnected within institutions, practices, beliefs, that shape the social determinants of health and health outcomes. An example of measurement might include the assessing how racial residential segregation is a conduit for observed microlevel behavioral outcomes. Applying this idea, one may consider including a measure of racial residential segregation and target its consequences, such as food deserts and limited access to services, as mechanisms for preventive interventions. This moves beyond traditional approaches, which often focus on neighborhoods void of system level contextual processes to explain behavioral outcomes. Moreover, Gee and Hicken (2021) offer insights on the need to revisit and rebuild preventive interventions, as “racial inequities in – health will persist until we redirect our gaze away from specific institutions and specific individuals and populations, and instead focus on the resilience connections among institutions and their racialized rules” (pg. 293).

To effectively measure systemic racism and the perpetuation of racial inequities, greater consideration needs to be given to expanding data sources that allows for identifying upstream factors and processes that affect health and well-being. In fact, census data collects information at county and state level across governmental entities about educational funding, employment patterns, and other factors that have implications for health and well-being. Furthermore, it is important to consider multiple measures of racism as a multifaceted determinant of health and to consciously consider these systems in the design and testing of preventive interventions.

## Advancing the Measurement of Toxic Waters of Racism Through Community Voice

We must rebuild metrics to assess health inequity and its determinants, particularly when guided by community voice. This is essential for all forms of prevention research: studies of risk and protective mechanisms, studies of program efficacy and effectiveness, and studies of dissemination and implementation, including revisiting and retooling methods to design and assess multilevel interventions to promote health equity by identifying and intervening on mechanisms that perpetually toxify the downstream flow of structural and systemic waters filled with pollutants that are commonly characterized as social determinants of health, including residential segregation, unfair lending practices, and environmental and social injustices, emitting numerous negative health consequences. For example, Adkins-Jackson et al. (2022) and Chantarat et al. (2021) developed a multidimensional measure of structural racism using latent class analysis and indicators such as police exposure, Black-White residential segregation, and inequities in Black-White income, education, criminal justice, employment, and home ownership. They then leveraged this measure to demonstrate the relationship between structural racism and health outcomes such as low birth weight and hypertension (Chantarat et al., 2022).

## Engaging in Inquiry to Understand Intersecting Forms of Oppression

Central to our discussion is the acknowledgement that systemic racism has historical legacies, is contemporaneously manifested through life-limiting social determinants of health and serves as a key driver of health disparities and inequities experienced by BIPILA populations. During the PSMG series, Lisa Bowleg called on prevention scientists to embrace critical frameworks such as intersectionality and critical race theory to center community voices and individual experiences within the context of these broader forces. Dr. Bowleg emphasized that prevention scientists should increase their competency in these critical frameworks so that they can conduct research that helps “reduce, not just document, health inequities among Black men.” As a demonstration of the transformative influence of using the intersectionality framework, Dr. Derek Griffith presented on how structural racism and the stress of COVID-19 intersect to concentrate diseases that then interact to create syndemics—two or more epidemics. These syndemics significantly increased the likelihood that Black men experience increased rates of heart disease, COVID-19 mortality, and other adverse health outcomes. Dr. Griffith’s presentation also emphasized the larger, historical, and current social contexts that elevates stress among Black men, such as how the “heightened visibility” that Black men adds stress to their daily lives as they code switch and modify their behaviors to appear less threatening and more capable than harmful stereotypes assign them. Through an intersectional reflection on US policy, Dr. Griffith elucidated how some policies only impact certain disease determinants, while leaving other determinants, such as structural racism, intact.

## Identifying Blind Spots in Prevention Science

Taken as a whole, presentations in the PSMG series led us to identify three “blind spots” in Prevention science. Blind spots are areas where views and perspectives are obstructed and not seen or inspected, hindering us from exercising judgment.

### Individualist Framework

A major blind spot in the field of Prevention Science is that our scientific studies have been primarily focused on developing and testing microlevel interventions, such as those delivered to the individual, family, or classroom, but void of targeting larger contextual factors and system-level processes. This is reinforced by existing service systems, including schools and public health agencies, that emphasize individual responsibility for health or education without attending to the social and economic conditions that increase health risk and disrupt individual resilience.

Despite successes in demonstrating preventive impact targeting micro systems (Mihalic & Elliott, 2015) effect sizes are often relatively small and heterogeneous, and effects are not always sustained (Sandler et al., 2014). In fact, programs that focus on building individual and family resilience often ignore the upstream environmental factors and processes that create and sustain chronic exposure to discrimination and systemic racism, or the social structures necessary to sustain change or reduce that exposure and its subsequent effect on health and well-being (Beard et al., 2022; Jones, 2000). This has limited efforts to develop intervention approaches that target community and larger societal systems (although see the work of Kellam and Langevin (2003) for a program that has demonstrated macro-level effectiveness.) Expanding our metaphor, lack of attention to multiple system level preventive interventions ignores mechanisms that create downstream toxic waters. For Prevention Science, the consequence of not addressing upstream issues is the development of interventions that provide some limited help in swimming against the current.

Restricted attention to microlevel systems is also found in disciplines that disproportionately focus on and invest in the translation of biomedical research, drug and technology development, research trial and recruitment, and other bench-to-bedside components of basic science translation to the exclusion of other basic sciences—such as epidemiology, behavioral science, psychology, communication, cognition, social marketing, economics, political science in the pursuit of translating novel, effective therapies (Woolf, 2008). This hyper-focus on individualist biomedical explanations and solutions can crowd out a fuller investigation of the interaction between the individual and the environment that is fundamental to racial health disparities. Joining basic science discoveries with a rigorous understanding of fundamental social processes made available through the applied social sciences should be considered conjointly in earlier phases of the translational biomedical research processes rather than after the innovation or technology has been developed and tested for efficacy.

#### Proposed Solution

Adapting Krieger’s theory of an “ecosocial approach” to population health for Prevention Science would necessitate embracing the perspective that “no aspect of our biology can be understood divorced from knowledge of history and individual and societal ways of living” (Krieger, 2001, p. 672). The benefit of applying an “ecosocial approach” in biomedical research translation might be indicated by the failure to reach Black and Brown populations with PrEP as an HIV prevention tool when compared to greater awareness and uptake among White MSM. An ecosocial approach would warrant conducting a sociological investigation alongside the biomedical translation, with profound involvement from key populations disproportionately impacted by and at high risk for HIV transmission. Moreover, translational research in prevention can avoid pathologizing health disparity populations by closely linking the discovery of biological bases and mechanisms with social processes and the environmental conditions that perpetuate disparities and social injustices. It may be that a richer understanding of mechanisms of social problems will reveal biological as well as socio-structural implications for the design and implementation of prevention interventions as they are coproduced (Woolf, 2008).

### Ignoring Heterogeneity

A second blind spot is the strong preference for designing studies that emphasize the highest level of experimental control at the expense of ignoring heterogeneity of impact and attention to more complex systemic factors that create and sustain toxic environments of discrimination and systemic racism. One consequence of ignoring heterogeneity is favoring narrowly defined interventions and implementation strategies over those that address the more complex and challenging research questions about how factors that cause and perpetuate social injustice and inequalities can be removed from the environments that adversely affect minoritized individuals and communities. Ignoring heterogeneity also leads to a dearth of attention not only to whether evidence-based interventions need to be tailored across populations, but also when and how successful implementation of tailored strategies improves the lives of those most affected by disparities (Brown et al., 2018).

#### Proposed Solution

We draw on several examples to offer guidance on how to address this blind spot. For example, the full knowledge-to-action approach*,* which includes implementation science, can be directed more towards equity (Singh, 2021). One potential solution is for prevention scientists to become more explicit in clarifying the hypothesized range of contexts and populations that their theories of risk and protection encompass and expanding those theories and intervention targets to include economic and social processes that sustain or reduce exposure to toxic environments. Moreover, a more explicit example was shared during a PSMG presentation, in which Singh encouraged us to include health equity in our frameworks that aim to characterize barriers to innovation implementation by presenting an updated version of the Health Equity Implementation Framework (Woodward et al., 2021). This framework emphasizes the need to consider equity within three key domains: the individual, patient-provider interactions, and the social context shaping individual health. Singh presented practical guidance for how these domains might impact our frameworks, interventions, and methods, including sample measures and methods that might align with each. For example, the Medical Mistrust Index, patient interviews, and document analysis were recommended to increase understanding of patient-level characteristics shaping health and innovation appropriateness for each population. Singh also presented a case example of how these health equity domains informed study methods to identify barriers and facilitators to service access and successful treatment of Hepatitis C Virus among Black American patients seeking care in the U.S. Department of Veteran’s Affairs. The interviews revealed numerous themes relevant for Prevention Science, including the need for providers to communicate their decision-making about the need for interventions, patient hesitancy to engage with providers who are racially biased, and societal stigma regarding Hepatitis C that led to lack of awareness and understanding of risk factors and treatment opportunities. This case study serves as a powerful example of how a focus on equity in our frameworks can increase understanding of how multi-level factors contribute to health and how these factors can be addressed through actionable strategies aligned to targeted populations’ needs and strengths.

Furthermore, research methods that capitalize upon the current knowledge base to address issues of heterogeneity are needed. Amuta-Jimenez et al. (2020), for example, offered a potential solution—synthesize findings in existing randomized trials to assess heterogeneity of impact and examine whether preventive interventions may differentially affect minoritized populations who are often underrepresented in these studies. Another strategy is to combine data from multiple studies to overcome under-representation of populations in existing trials (Brown et al., 2018). Another potential solution is to deliberately extend an ongoing preventive intervention project that initially involves few minoritized individuals with a project that extends the inquiry to underrepresented populations. This will necessarily require adaptation (e.g., ADAPT-ITT), but there are methodologic approaches that allow for borrowing strength from existing studies (Aarons et al., 2017). A final suggestion is to invest in more longitudinal research studies specifically designed to address structural and systemic racism and implications for documenting long-term developmental effects and consequences on and in minoritized communities, from pre-conception to aging, such as critically teasing out factors that explain high mortality rates among Black mothers and infants, soaring rates of suicide among middle school age Black youth, to name a few (Kellam & Langevin, 2003; Murry et al., 2007, 2019; Prado & Pantin, 2011). Longitudinal preventive intervention research studies are far fewer in percentage than the proportion of Blacks or Latino/s in the USA. It is critical that more of these studies are initiated promptly (Buckley et al., under review), given the pervasive nature of systemic racism and its consequences for minoritized families, children, and communities.

### Ignoring Voices

The final blind spot focuses on the ready acceptance and expectation that biases Prevention Science projects to commence and be sustained through the involvement of communities and organizations that are already relatively rich in capacities and resources, while failing to include BIPILA individuals, communities, and entities less well-resourced. Despite efforts to promote diversity, equity and inclusiveness in academic institutions, professional societies, and federal policies, the field of Prevention Science continues to be dominated by racialized hegemonies reified through the disproportionate training of and resource availability to White investigators. Those conducting studies are often underrepresented by Black, Latino/a/x, and indigenous populations. This is true of randomized efficacy/effectiveness trials of prevention programs (Perrino et al., 2015), and is even more pronounced in studies of implementation (Benbow et al., 2020).

#### Proposed Solution

To address this blind spot, we recommend that prevention science revisit the fundamental knowledge bases and repositories of evidence-based prevention theory. This process can begin with reimagining and making space for alternative ways of “knowing” and “doing.” For example, consider the cultural relevance of language in the use of such terms as “best practice.” *Best practices* often assume that Western-based knowledge and experiences with given practices can be universally applied to other cultures (Wesley-Esquimaux & Snowball, 2009). In distinction, “wise practices” (Wesley-Esquimaux & Snowball, 2009, p. 390) are idiosyncratic, contextual, textured, and culturally relevant ways of “knowing” and “doing” that align with community norms, principles, tools, and decisions that communities have already developed to navigate challenges associated with “swimming in toxic waters.”

Another recommendation is to address the results of the NIH commission report (Hoppe et al., 2019), which documented persistent funding disparities in the success rates for grants supporting minoritized scientists, Black scientists, in particular, by ensuring that there is greater *representational equity* in federal funding. Addressing this persistent problem not only provides another mechanism to amplify the voices of BIPLA in prevention science but also increases representational equity in the number of projects led by BIPLA investigators. This strategy may hold promise for increasing diversity of perspectives and theories of ways of knowing, and in turn, facilitating a paradigmatic shift by disrupting racialized hegemonic strongholds of prevention knowledge and practice. This will also require deliberate, systematic governmental and institutional infrastructure to monitor, with accountability that the preponderance of white investigators conducting preventive intervention trials in minoritized communities is no longer the *standard way of operating*. Ultimately, these and other upstream focused practices and policies designed to engage and include the voices of minoritized researchers, practitioners, families, and communities in health equity research initiatives, hold the potential to advance Prevention Science. Evidence of our advancement will be measured in terms of the extent to which the work produced from our efforts demonstrates improvement in human condition through reductions in disparities, including but not limited to mental health, academics, drug/substance use, and justice involved youth. In addition, we will evince greater changes in the promotion of a more just and equitable enrollment of BIPILA communities and families in preventive intervention research studies, led by BIPILA research scholars.

## Call to Action and Questions to Ponder

Addressing systemic racism through Prevention Science will require shifting our scientific focus more towards impactful higher level or upstream environments, both through theoretical framing and retooling, re-examining the four core methodologic foundations that Prevention Science has so long relied on to establish evidence—design, measurement, modeling, and efficacy and effectiveness testing. We offer insights on ways to begin the retooling and rebuilding process by summarizing ideas emerging from the PSMG Series.

### Re-examining Research Designs in Prevention Science

One critical challenge is that the traditional individual-level randomized trial is not appropriate when considering interventions that change larger contexts. For example, a comparison of the statistical power for a classroom-based intervention, with 30 individuals, compared to one directed at the whole school, can easily require an order of magnitude larger study to achieve the same statistical power (Brown & Liao, 1999). It is quite possible to conduct larger implementation trials, say at the county level, using two levels of county randomization: one being time of intervention, using a stepped wedge design, and the second being random assignment to one of two alternative implementation strategies (Brown & Liao, 1999). Setting one of the implementation strategies to provide a unique focus on overcoming disparities can lead to a direct test of this adaptation against that provided by a standard condition. We also consider complementing our design choices with studies that may need limited or no randomization. For example, natural experiments could be leveraged when a state or health system decides to scale up the delivery of an intervention and researchers collect relevant information on equity using the Health Equity Implementation Framework (Woodward et al., 2021). Such studies can be enhanced through careful use of regression discontinuity designs (Trochim, 1990; Stuart, 2007; Mercer et al., 2017; Knapp et al., 2019; Musci & Stuart, 2019) and engaging in internal reflective evaluation of Prevention Science approaches and strategies that may occasion acknowledging scientific blind spots that inform and guide our work, that foster and perpetuate inequities.

### Re-tooling Measurement and Approaches

When considering evidence of replication and intervention effectiveness, we must also include measures to assess systemic racism and inequities as preventive intervention targets, with a focus on intervening through critical systemic processes to promote equity. Furthermore, developing metrics to harmonize data across preventive intervention studies is also warranted, suggesting the need to revisit and refine theories, measures, and thresholds for establishing efficacy and effectiveness. To jumpstart the process, Buckley and Hill urged the field of Prevention Science to move beyond simple effect sizes and consider contextual impacts of interventions upon inequities as evidenced by advancement in equity.

### Re-building efficacy and effectiveness testing in Prevention Science

Common, formal definitions of evidence-based interventions note the importance of replicated, rigorous intervention trials that demonstrate efficacy and effectiveness (Gottfredson et al., 2015; Mihalic & Elliott, 2015). However, an emphasis on effectiveness for diverse populations is often lacking, often treats broad racial/ethnic subgroups as homogenous, and ignores the impact of contextual factors such as systemic racism on program effectiveness. Specification of hypothesized moderators and mediators that are or of particular relevance for BIPILA communities, such as how structural racism are experienced, taught about or prevented (i.e., through deployment of methodologies such as Public Health Critical Race Praxis; see Ford & Airhihenbuwa, 2010; Ford et al., 2018), should be part of the research investigation and replication process.

Buckley and Hill, in their presentation to PSMG (Buckley & Hill, 2021), offered important insights on the need to refine the notion of evidence in prevention trials research. The scholars described a recent critical review of the Blueprints Registry for Healthy Youth Development, a repository of “experimentally proven” programs, to revisit the equitability of the registry content. The premise of their discussion was the recognition of important challenges to the field that includes whether “evidence-based” means understanding whether an intervention will work without culturally responsive adaptation, or whether unique interventions are required to equitably improve health for each community. The transportability of parenting interventions across nation states, for example, has been supported by meta-analyses (Gardner et al., 2016). Guttmannova et al. (2017) found similar risk and protective factors between rural, predominately White youth and indigenous youth in the USA and Canada in a database of *Communities that Care* surveys.

Shared risk factors might not translate to shared intervention targets or outcomes, however. Few studies have evaluated the transportability of interventions across cultures and communities, following a common assumption that interventions validated in White communities can be recommended for all populations without qualification. The Blueprints team is conducting a review to examine the representation of racial and ethnic groups in prevention interventions, and the extent to which outcomes are reported for these groups. Relatedly, the Blueprints evidence standards now emphasize that the populations for which an intervention is intended should be clearly specified. Buckley and Hill proposed that, from the researchers’ perspective, a motivating question could be: “How can we ensure that our intervention is anchored in justice to produce the most positive, equitable impacts for each community who elects to adopt it?” While policy makers might ask: “How can we know that we are funding and implementing the most effective programs to promote equity and justice in for our communities?”.

Buckley and Hill also discussed a need for being more precise in prevention science’s language when reporting that an intervention is “effective” or “evidence-based.” Greater specificity is needed about whether interventions are effective for specific: (1) outcomes, (2) populations, (3) settings, and (4) timeframes—analogous to a precision medicine approach for high disparity populations. Intervention effects should be precisely described in terms of their range of application. For example, it is not sufficient to report that a COVID-19 vaccine is 95% effective, as this statement is not only ambiguous, but the lack of specification might garner discomfort and distrust in the scientific evidence or inherent uncertainty of the intervention. An alternative, equity-conscious statement of effectiveness could read: “A COVID-19 vaccine is 95% effective at reducing hospital admissions among a racially/ethnically representative sample of US adults aged 18–30.”

Even when a high level of evidence regarding effectiveness exists, Supplee noted that research is less likely to be used when there is a high degree of polarization among decision makers. Understanding and navigating complex social dynamics will be critical to the Prevention Science evidence generation and translation process and cannot be considered an optional action or one to be addressed by others once an intervention has been developed and tested. Doucet reminded us, “Research is inextricably implicated in the societal structures and systems that have served to maintain power hierarchies and accept social inequity as a given.” As such, the presenters noted that our goal in Prevention Science should not be pure objectivity or neutrality, akin to colorblind racism, but a scientific group that critically interrogates how deficit discourses arise, are perpetuated, and can be dismantled through science and practice.

### Re-envisioning Prevention Scientific Inquiry Through BIPILA Communities’ Engagement

A second major theme involved deeper attention to partnering with BIPILA communities, in line with positions by the World Health Organization that it is essential in developing new paradigms in Prevention Science to “…realize [that] health equity requires *empowering people*, particularly socially disadvantaged groups, to exercise increased collective control over the factors that shape their health (Marmot et al., 2021).” Pursuing equity in and through Prevention Science demands a fundamental reorientation of our scientific enterprise’s relationships with minoritized communities. There is a need for an internal interrogation and reframing of “whose interests are being voiced and whose are being served” (Petiwala et al., 2021). This ethic of inclusion should be present throughout the scientific inquiry and evidence derivative processes. Furthermore, the voices of those who are experiencing a disproportionate burden of disease and who have been exposed to profound barriers in overcoming risk factors for poor health outcomes should be given priority. This is necessary to capture what matters more fully, in terms of how, when, and for whom prevention programs are developed. It also needs to be pursued throughout all stages of design, development, and implementation of preventive interventions. Yamin (1996) endorses this viewpoint by emphasizing that, “A right to health based upon empowerment [implies] that the locus of decision making about health shifts to [and by engaging] the people whose health status is at issue” (*p*. 407). While community engaged research perspectives and community advisory boards are common strategies in Prevention Science, the application of a human rights-based approach offers innovative ways to broaden and intensify community voices to facilitate engagement throughout the “full translational spectrum of the Prevention Science model” (Fishbein et al., 2016, *p*.5). This approach will require letting go of the reins of power and trusting the process of community engagement, including reexamining the notion of culturally tailoring preventive interventions. Engaging and integrating community voices in research can also increase community commitment to the project (Israel et al., 1998; Spalluto et al., 2019), leading to more complete project adoption and better sustainment. Insiders’ perspectives can ground research questions in important social and political contexts essential to designing effective intervention responses that address the root causes and consequences of systemic racism on health and well-being.

Calancie et al. (2021), in a systematic scoping review of health cross-sector collaborations, identified several terms that have been used to describe the construct of community voice and concluded that the concept is inclusive of mechanisms that amplify silenced realities and perspectives of subaltern groups. Centering community voices in the preventive intervention research process can also facilitate the mobilization of collective or shared power in decision-making to shape the overall discourse and production of Prevention Science knowledge and translation efforts (Petiwala et al., 2021). This will aid Prevention Science in revising and refining research practices and policies that are embedded in the science and institutions that have, at times, enshrined the power, values, and structures that impede social justice and health equity for populations who are the recipients of prevention programs.

This investment may also enhance communities’ sense of control, thereby addressing historical mistrust, as communities are given place and space to integrate their lived experiences in the research process. This process can uncover hidden problems that communities encounter, as well as untapped protective processes evolving from grassroot efforts to navigate toxic waters, increasing the likelihood that preventive interventions are ecologically, socially, and culturally relevant and responsive. In addition, the centering of communities’ voices in Prevention Science research offers opportunities to create counter-narratives beyond deficit scientific discourses that pathologize marginalized communities and perpetuate hegemonic standpoints that are often restrictive and hinder scientists’ ability to intervene from the perspectives and contexts of BIPILA peoples. It is important to recognize the unique strengths, to characterize those strengths and use those strengths to design preventive interventions.

The theoretical and empirical foundations of Prevention Science have often been thought of as strength-based, emphasizing the development of individual competencies and resilience. A more comprehensive strength-based framework would also attend to protective social mechanisms at the level of the family, school, neighborhood, or community, and how those can be leveraged to challenge systemic racism and discrimination at those levels. One prevention strategy that has seen little development is to adapt our preventive interventions to build on community-specific strengths, rather than deliver interventions that have not been designed to enhance those strengths within minoritized communities. For example, African Americans are less likely to receive COVID-19 vaccines (Center for Disease Control & Prevention, 2022) and more likely to die from COVID-19 than are whites (Centers for Disease Control & Prevention, 2022). For example, a major source of COVID-19 transmission is through contact with individuals residing in the same home who are infected with the virus (Cerami et al., 2021). Given this, greater consideration should be given to identifying, designing, and developing effective ways to engage and protect all the adults in a household rather than focusing on changing behavioral patterns at an individual level. Using a family-centered approach would be particularly beneficial for BIPILA families, who, because of their cultural configurations of the household, are more likely than whites to live in multigenerational households (Simpson et al., 2021). In addition, a strengths-enhanced prevention strategy centering and empowering African American women, who uniquely hold a central and powerful role in their family’s health and the community more broadly, could serve as another potential strategy to enhance vaccination acceptance and completion among members of their household. For example, by eliciting their feedback about effective ways they can serve as a family health promotion and preventive intervention change agent with ample support and resources to fulfill this critical role. To illustrate this point, we draw on the statement of an African American mother, “Even if my family doesn’t trust the system, they trust me to have done the research to make sure that the vaccine is safe.” She is a primary source of family health care and perhaps to be considered, with no less significance, than the primary health care provider. Moreover, this emphasis elaborates the importance of engaging trusted community members and including their voice in the design, development, and implementation of preventive interventions. Furthermore, preventive intervention strategies and approaches that emerge from partnering with trusted community leaders, especially through their contributions to informing epistemologies of prevention science is an intervention that may, in fact, also engender trust. Establishing trust is key to engaging African Americans in research and preventive interventions, given the detrimental consequences of the historical abuses of African Americans by scientists (e.g., the Tuskegee Syphilis Study (Reverby, 2001).

### Re-examining the Complexities of Advancing Health Equity in Prevention Science

A third major theme reflected the insight that racism operates in complex multilevel systems, both within and between subsystems. It is reinforced by structures, policies, programs, and practices that produce privilege and advantage to its perpetrators. That people live in environments composed of multiple systems—including social, physical, environmental, economic, and political systems—demonstrates the need for designing preventive interventions that target upstream, middle stream, and downstream systems that eventually filter into the lives and experiences of individuals, families, and communities. Complex systems act and interact through feedback loops that produce emergent outcomes and behaviors within themselves and as consequences of their interactions (Meadows, 2008). To alter these emergent outcomes, the ways in which system components interconnect dynamically over time must be identified and targeted (Sterman, 2001).

Theories of complex systems, such as those put forth by systems science and socio-ecological models (Sterman, 2001; Bronfenbenner, 1995; Jayasinghe, 2015), provide valuable frameworks and methodologies to deploy in understanding, assessing, and addressing systemic racism and its consequences for social justice and health equity. This approach allows for an integrated and holistic approach in the design and testing of preventive interventions. Desmond’s (2018) explanation of anti-poverty efforts in the USA offers insight on the need for the use of complexities theories to address social determinants of health: “But when we shrink the problem, the solution shrinks with it; when small solutions are applied to a huge problem, they don’t work; and when weak antipoverty initiatives don’t work, many throw up their hands and argue that we should stop tossing money at the problem altogether. Cheap solutions only cheapen the problem” (p. 36).

Thus, programs that focus on enhancing individual resilience may provide Black communities with a life raft in the midst of toxic waters, helping them cope with oppression, but this *does not solve the problem of racism in the USA*. Applying complex systems approaches holds potential and promise to address health equity through preventive interventions targeting key drivers and entry points where structural racism pollutes waterways and maintains its hold in our systems. In addition, using this approach we can identify service delivery systems that provide opportunities for prevention intervention, such as the economic, justice system, health care, and educational institutions. There is, therefore, a need to incorporate systems science into prevention research frameworks both to enhance our knowledge of how environments, composed of multiple subsystems, interact to create, sustain, or amplify systemic racism, and to then identify and test strategies for their efficacy and effectiveness at fostering optimum subsystem and overall system health and well-being and equitable outcomes. For example, the city of Baltimore incorporated complex systems science thinking and simulation modeling to understand the numerous complex systems factors involved in the City’s obesity epidemic (National Academies of Sciences, Engineering, & Medicine, 2021). This initiative led to a multiscale, multi-intervention project focused on Baltimore City’s food system and the health, economic, and environmental disparities in healthy food priority areas (National Academies of Sciences, Engineering, & Medicine, 2021).

Several systems science tools are available that can be adopted and optimized. For example, agent-based modeling can examine how preventive implementation strategies address the unique needs of BIPILA communities by examining the interactions between individuals and between individuals and their environments to explore how health challenges are exacerbated or resolved (McNulty et al., 2019; Vermeer et al., 2021). In addition, advances have been made in embedding systems science methods within theoretical frameworks that advance health equity and social justice. For example, Frerichs et al. (2016) offered guidance on how to integrate critical race theory with group model building methods, a participatory form of system dynamics modeling, for addressing community violence and enhancing the cultural responsiveness of this modeling approach and the models it generates. They proposed that “incomplete mental models and implicit biases due to race likely contribute to the debate that often surrounds discourse on racial disparities in violence” (p. 2). Their work underscored the importance of attending to community voices and the complexities of participants’ lived experiences when designing health equity research and preventive intervention trials. To move beyond a desire to reduce disparities, but, instead, advance health equity will require revisiting Prevention Science’s biases and mental models within the context of historical oppression that shapes contemporary social debates.

As key influencers of health promotion, Prevention Scientists are positioned to address and advance health equity by revisiting whether we are implicitly or explicitly perpetuating inequity or facilitating health equity. By recognizing and deliberately acknowledging that racism manifests itself as a toxic environment within which BIPILA populations are forced to reckon with daily, there is an urgent need to critically evaluate the extent to which our own policies and practices may contribute to and perpetuate levels of toxicity. We view the role of Prevention Science, appropriately restructured, as a potentially powerful tool to promote equity. There are a diverse set of strategies that have been developed to address environmental changes to improve public health. We reflect on two environmental public health interventions to illustrate this point. One example is John Snow’s removal of the Broad Street water pump handle to remove the source of cholera, and more recently the belated changes in Flint Michigan to protect against lead contamination in the water supply (Tulchinsky, 2018). These were far more effective than any strategy that focused on individuals or households alone. Prevention Science can have greater impact on equity by expanding work on environmental interventions to address the pernicious effects of racism. Recognizing this, SAMHSA has prioritized the role of environmental interventions to reduce underage drinking (U.S. Department of Health and Human Services, Substance Abuse and Mental Health Services Administration (SAMHSA), Interagency Coordinating Committee on the Prevention of Underage Drinking, 2018).

We conclude with a set of discursive questions that prevention scientists must be occupied with in our efforts to advance health equity as posed by Nelly Williams, a community activist invited to present to the PSMG network:

What actions are you taking and willing to take? Will you include restorative justice as a way forward? How deep are we willing to go to mend the wrongs and level the playing field for all who have suffered the blows and burden of racism and discrimination alive and thriving in our society today, impacting all institutions that BIPILA rely on to meet their essential needs? Are you willing to allow yourself to be informed and educated by those who are most impacted by systemic racism? Are you willing to develop a unified approach to building trust between community members, research institutions? Are you willing to invest in hope building strategies to lift up the burden and heal the pains of systemic racism that have generational consequences of BIPILA? Will you meet me where I am, and when you do can you face the music? When will you say that enough is enough and do something about it? (Murry et al., 2020)

We have attempted to craft a response to those questions, though the proof of concept, or the feasibility of our answers becoming a reality depends on the extent to which the field of Prevention Science is willing adhere to the recommendations and call to action. Thus, we conclude by emphasizing that our aim is not just to celebrate the extraordinary strength and resilience of BIPILA but to also *recognize* and *require* transparency in funding practices to improve and increase *representational equity*, using a social justice and equity framework to consciously evaluate who receives funding and who serves on review panels. In addition, we urge prevention science researchers and practitioners to boldly find ways to address and change the fundamental structural inequities that stand in the way of addressing structural racism and promoting health equity. As scholars with institutional power, we are in a position to lay a foundation to be a forerunner of centering health equity research and practices in the field of prevention science, and in so doing can inform and guide other disciplines. Such paradigmatic shift in our field can serve to directly or indirectly provide a platform to translate our research to reconstructed upstream policies that perpetuate oppression, structural and systematic racism, to instead advance equities to improve the health and well-being of those disproportionately impacted by these toxic downstream waterways. A place to begin is to answer the questions posed by community member whose everyday life experiences: When will [we] say *enough and do something about it?*
