# Correction: Re-envisioning, Retooling, and Rebuilding Prevention Science Methods to Address Structural and Systemic Racism and Promote Health Equity

**DOI:** 10.1007/s11121-024-01701-x

**Published:** 2024-06-26

**Authors:** Velma McBride Murry, Cory Bradley, Gracelyn Cruden, C. Hendricks Brown, George W. Howe, Martín-Josè Sepùlveda, William Beardslee, Nanette Hannah, Donald Warne

**Affiliations:** 1 Departments of Health Policy & Human and Organizational Development, Vanderbilt University, Nashville, TN USA; 2 Washington University School of Medicine in St. Louis, St. Louis, MO USA; 3 Oregon Social Learning Center, Eugene, OR USA; 4 Northwestern University Feinberg School of Medicine, Chicago, IL USA; 5 George Washington University, Washington, DC USA; 6 Florida International University, Miami, FL USA; 7 Harvard Medical School, Boston Children’s Hospital, Judge Baker Children’s Center, Boston, MA USA; 8 Center for Indigenous Health, Johns Hopkins University, Baltimore, MD USA

**Correction to: Prevention Science (2022) 25:6-19** 10.1007/s11121-022-01439-4

The article “Re-envisioning, Retooling, and Rebuilding Prevention Science Methods to Address Structural and Systemic Racism and Promote Health Equity”, written by Murry, V.M., Bradley, C., Cruden, G., Brown, C.H., Howe, G.W., Sepùlveda, M-J., Beardslee, W., Hannah, N., and Warne, D., was originally published Online First without Open Access. After publication in volume 25, issue 1, page 6–19 the author decided to opt for Open Choice and to make the article an Open Access publication. Therefore, the copyright of the article has been changed to © The Author(s) 2022 and the article is forthwith distributed under the terms of the Creative Commons Attribution 4.0 International License, which permits use, sharing, adaptation, distribution and reproduction in any medium or format, as long as you give appropriate credit to the original author(s) and the source, provide a link to the Creative Commons licence, and indicate if changes were made. The images or other third party material in this article are included in the article’s Creative Commons licence, unless indicated otherwise in a credit line to the material. If material is not included in the article’s Creative Commons licence and your intended use is not permitted by statutory regulation or exceeds the permitted use, you will need to obtain permission directly from the copyright holder. To view a copy of this licence, visit http://creativecommons.org/licenses/by/4.0/.

The original article has been corrected.

